# HB-GAM (pleiotrophin) reverses inhibition of neural regeneration by the CNS extracellular matrix

**DOI:** 10.1038/srep33916

**Published:** 2016-09-27

**Authors:** Mikhail Paveliev, Keith K. Fenrich, Mikhail Kislin, Juha Kuja-Panula, Evgeny Kulesskiy, Markku Varjosalo, Tommi Kajander, Ekaterina Mugantseva, Anni Ahonen-Bishopp, Leonard Khiroug, Natalia Kulesskaya, Geneviève Rougon, Heikki Rauvala

**Affiliations:** 1 Neuroscience Center, University of Helsinki, Finland; 2 Neuroscience Institute Marseille, France; 3 Neuroscience and Mental Health Institute, Faculty of Rehabilitation Medicine, University of Alberta, Edmonton, Canada; 4 Institute of Biotechnology, University of Helsinki, Finland

## Abstract

Chondroitin sulfate (CS) glycosaminoglycans inhibit regeneration in the adult central nervous system (CNS). We report here that HB-GAM (heparin-binding growth-associated molecule; also known as pleiotrophin), a CS-binding protein expressed at high levels in the developing CNS, reverses the role of the CS chains in neurite growth of CNS neurons *in vitro* from inhibition to activation. The CS-bound HB-GAM promotes neurite growth through binding to the cell surface proteoglycan glypican-2; furthermore, HB-GAM abrogates the CS ligand binding to the inhibitory receptor PTPσ (protein tyrosine phosphatase sigma). Our *in vivo* studies using two-photon imaging of CNS injuries support the *in vitro* studies and show that HB-GAM increases dendrite regeneration in the adult cerebral cortex and axonal regeneration in the adult spinal cord. Our findings may enable the development of novel therapies for CNS injuries.

Chondroitinase ABC (ChABC) treatment has been extensively used to demonstrate that the CS side chains of CSPGs are potent inhibitors of regeneration and plasticity in the extracellular matrix (ECM) of brain and spinal cord^1^,^2^,^3^,^4^,^5^,^6^,^7^. We have sought for less invasive ways to enhance CNS regeneration and plasticity, which might be useful for the development of therapies for CNS traumas. To this end, we considered endogenously occurring molecules that might modulate functions of the CNS matrix in the juvenile brain which displays high plasticity in comparison to the adult brain.

HB-GAM/pleiotrophin was initially isolated as a heparin-binding neurite outgrowth-promoting factor for central neurons^8^,^9^. Its expression peaks during the first 3–4 weeks of postnatal development in rat brain^10^ corresponding to heightened plasticity of the juvenile brain^11^. The expression level at this stage is very high, up to 10–15 μg/g of wet tissue weight^8^, which might be sufficient to modulate matrix structures^12^ that typically inhibit neural plasticity and regeneration. HB-GAM is secreted from neurons and glial cells upon cleavage of a classic-type secretion signal^10^, binds CS side chains of CSPGs with nanomolar K_d_ values^12^,^13^,^14^, and lines nearly all fiber tracts of the early postnatal rat brain^15^. Furthermore, the growth factor Midkine, which displays homology with HB-GAM, was recently reported to partially overcome CSPG inhibition of neurite extension^16^. Taken together, these studies suggest HB-GAM as a candidate molecule to modify interactions of CNS neurons with inhibitory ECM structures such as CSPGs. However, the possibility that HB-GAM could overcome or even reverse the inhibitory effects of the ECM on growth and regeneration of neurites has not previously been explored.

## Results

### HB-GAM promotes neurite outgrowth on CSPG substrates

**Figure 1:**
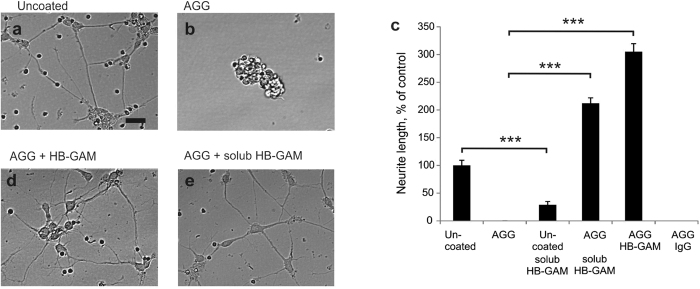
HB-GAM turns the CSPG aggrecan to a potent activator of neurite growth. (**a**–**c**) Neurite outgrowth in cortical neurons on substrates coated with aggrecan (AGG), aggrecan + IgG or aggrecan + HB-GAM. Neurons display poor attachment and no neurite growth on aggrecan (10 μg/ml) or aggrecan + IgG (both at 10 μg/ml)-precoated substrate. (**c**,**d**) In contrast, HB-GAM precoated together with aggrecan (both at 10 μg/ml) induces neurite growth (150–200 μm/neuron) compared to unprecoated plastic, aggrecan or aggrecan + IgG. (**c**,**e**) HB-GAM (10 μg/ml) added to the culture medium induces robust neurite outgrowth on the substrate coated with aggrecan but even inhibits neurite outgrowth on uncoated substrate. The scale bar in a (20 μm) is valid for (**a,b,d,e**). Error bars represent standard error of mean (SEM), symbols *, ** and *** represent P < 0.05, 0.01 and 0.001, respectively. All data are based on 3 independent experiments.

We first studied the effect of HB-GAM on neurite growth from primary CNS neurons plated on aggrecan, a major CSPG in the CNS^17^. As expected, the aggrecan matrix effectively prevented neurite outgrowth from the CNS neurons (Fig. 1a–c). Coating of HB-GAM together with aggrecan overcame the inhibitory effect (Fig. 1c,d). Moreover, delayed addition of HB-GAM to culture media promoted neurite extension from the primary CNS neurons already inhibited by aggrecan (Supplementary Fig. S1).

HB-GAM alone, added in solution at the time of cell plating on uncoated tissue culture plastic, did not promote neurite outgrowth, but rather displayed some inhibitory effect (Fig. 1c). Conversely, when HB-GAM was added in solution with the cells on aggrecan-coated wells, a prominent neurite outgrowth response was observed (Fig. 1c,e). HB-GAM did not act by reducing the amount of aggrecan coated on the substrate (Supplementary Fig. S2a). As an alternative to prepare aggrecan substrates, we used biotin-avidin interaction to firmly bind aggrecan to the culture wells. Inhibition of neurite outgrowth by aggrecan and the reversing effect by HB-GAM were observed in the same manner as in assays where aggrecan was simply coated on the culture wells (Supplementary Fig. S2b). The unexpected finding that the CSPG culture substrate enhances neurite growth in the presence of soluble HB-GAM does not therefore depend on the method used to bind the CSPG on the culture wells.

**Figure 2:**
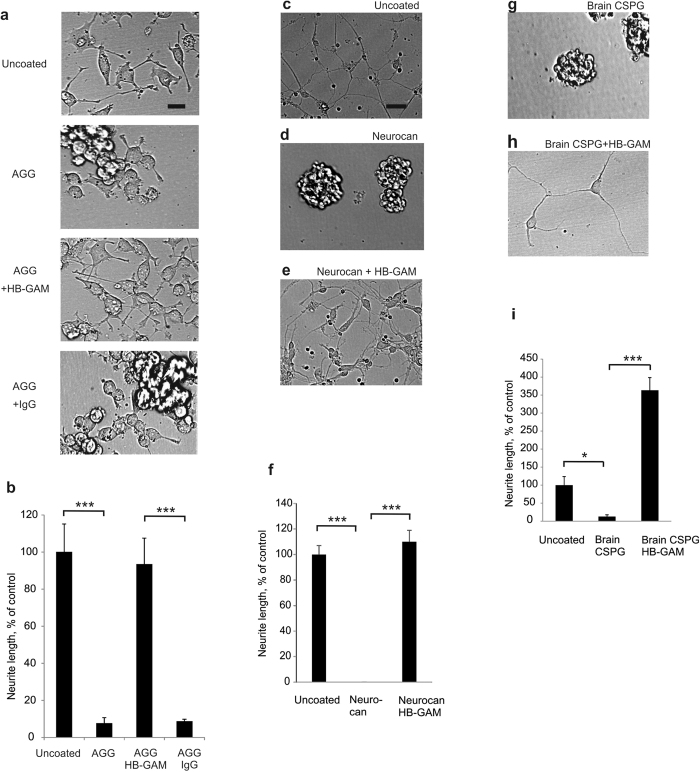
HB-GAM overcomes the CSPG-dependent inhibition of neurite growth in PC12 cells. HB-GAM overcomes the inhibitory effect of neurocan and brain-derived CSPGs on neurite growth. (**a**) PC12 cells plated on unprecoated culture plastic, on the culture plastic precoated with aggrecan, with aggrecan + HB-GAM or with aggrecan + IgG. Immunoglobulin G (IgG) was used as an unspecific control in precoating. All samples were treated with NGF (100 ng/ml). (**b**) Neurite length measured in live cell images obtained 1 day after plating. (**c**–**f**) Cortical neurons cultured on different substrates in the absence or presence of HB-GAM. Neurons were cultured on unprecoated plastic, on neurocan (5 μg/ml) or on neurocan (5 μg/ml) + HB-GAM (25 μg/ml). (**g**–**i**) Hippocampal neurons from E17 rats were cultured on the substrate precoated with brain-derived CSPGs (50 μg/ml) or with brain-derived CSPGs (50 μg/ml) + HB-GAM (10 μg/ml) or on the substrate without precoating (uncoated in panel i). Live cells were imaged after 36 h in culture. The scale bar (20 μm) shown in a and c is valid for (**a**,**c**–**e**,**g**,**h**). One way ANOVA was used.

The effect of HB-GAM on the CSPG substrate is not restricted to primary CNS neurons. A similar effect was observed with PC12 cells cultured in the presence of nerve growth factor (NGF), which by itself was not able to promote neurite outgrowth on aggrecan (Fig. 2a,b). To examine whether the HB-GAM effect on the matrix side is restricted to the CSPG aggrecan, we tested neurocan (another major CSPG of the matrix)^17^ and a CSPG mixture prepared from brain in the same assays as aggrecan. HB-GAM was also found to overcome their inhibitory effects on neurite outgrowth (Fig. 2c–i). The HB-GAM effect on neurite outgrowth on the CSPG substrates may therefore depend on its interaction with the CS side chains of the CSPGs.

### HB-GAM-induced neurite outgrowth on CSPG substrate depends on the CS chains

In agreement with the generally held view that the CS chains of the CSPGs are responsible for the inhibitory effect on regeneration and plasticity^1^,^2^,^3^,^4^,^5^,^6^,^7^, we observed that digestion of the CS chains of the substrate-bound aggrecan with chondroitinase ABC reduced inhibition of neurite outgrowth (Supplementary Fig. S3).

**Figure 3:**
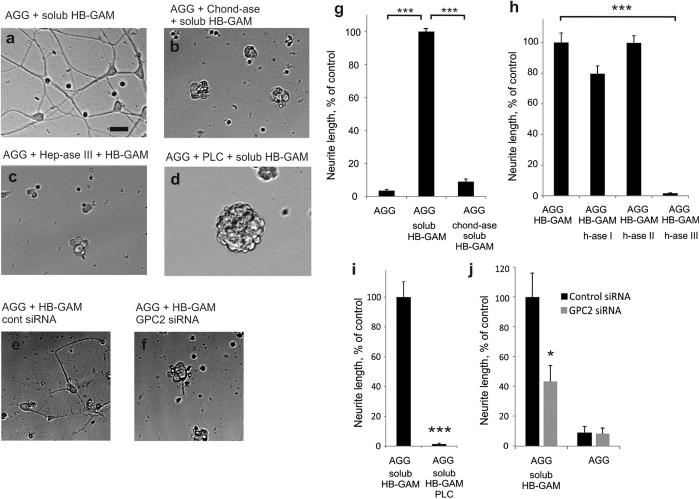
HB-GAM-induced neurite outgrowth on aggrecan depends on the CS chains of the substrate-bound aggrecan and on the HS chains of the neuron surface glypican-2. (**a**,**b**,**g**) Neurite growth induced by aggrecan + soluble HB-GAM is blocked by the chondroitinase ABC (2 U/ml, 30 min) pretreatment of the aggrecan-coated substrate. (**c**,**h**) Neurite growth on the aggrecan + HB-GAM subsrate is blocked by heparinase III (0.017 U/ml) but not by heparinases I or II (0.017 U/ml). (**d**,**i**) Neurite growth induced by aggrecan + soluble HB-GAM is blocked by preincubation of cells with phospholipase C (PLC; 0.4 U/ml, 1 h). (**e**,**f**,**j**) Neurite growth on aggrecan in the presence of HB-GAM is inhibited by the glypican-2 (GPC2) siRNA. The scale bar in a (20 μm) is valid for **a**–**f**. Error bars represent standard error of mean (SEM), symbols *, ** and *** represent P < 0.05, 0.01 and 0.001, respectively. All data are based on 3 independent experiments.

Another striking effect of chondroitinase ABC treatment was to abrogate the ability of aggrecan to enhance neurite outgrowth in the presence of soluble HB-GAM (Fig. 3a,b,g). These experiments show that the same CSPG can serve as an inhibitor of neurite outgrowth in the absence of HB-GAM or as an activator of neurite outgrowth in the presence of HB-GAM. The CS side chains of CSPGs are required to bind HB-GAM, and the resulting CSPG/HB-GAM substrate then induces neurite extension. This places HB-GAM as a pivotal factor to reverse the role of the CS chains in neurite outgrowth.

### Glypican-2 of the neuron surface is required for HB-GAM-induced neurite outgrowth on CSPG substrate

The CS chains might act through binding HB-GAM and presenting it to a cell surface receptor of neurons. Neurite outgrowth induced by HB-GAM has previously been shown to depend on binding to heparan sulfate (HS) chains of the neuron surface^15^. We therefore tested whether the HS chains located on the extracellular surface of neuron cell membranes are required for neurite outgrowth on aggrecan in the presence of HB-GAM. Of the heparinases tested, heparinase III (the heparinase that cleaves preferentially HS instead of highly sulfated heparin-type chains) caused a nearly complete inhibition of neurite outgrowth on aggrecan in the presence of HB-GAM, whereas heparinases I and II had little or no effect on neurite outgrowth (Fig. 3c,h). The HS proteoglycan syndecan-3 (N-syndecan) has previously been reported as an HB-GAM receptor^18^. We therefore performed experiments using syndecan-3 knockout neurons. No difference was observed in neurite outgrowth between the wild-type and syndecan-3 knockout neurons (Supplementary Fig. S4a–e). Therefore, syndecan-3 is not the heparinase III-sensitive HB-GAM receptor mediating neurite outgrowth on aggrecan.

We next carried out an unbiased search of putative HB-GAM receptors and associated components by incubating cultured CNS primary neurons with HB-GAM-coated magnetic beads. After lysis, the transmembrane fraction coupled to the beads was collected by magnets, and analyzed by liquid chromatography-mass spectrometry (LC-MS). Among the hits with high scores, glypican-2 was repeatedly found as the only cell surface component whereas the rest of the hits were intracellular components that probably include molecules involved in HB-GAM-induced cell signaling in neurons (Table 1). Glypican-2 is an HSPG attached to the cell membrane by a glycosylphosphatidylinositol (GPI) anchor. Therefore, the finding that HB-GAM-induced neurite outgrowth on aggrecan is abrogated by heparinase III is consistent with the view of the HS proteoglycan glypican-2 acting as a receptor.

We also tested the effect of phospholipase C, an enzyme that cleaves GPI anchors, on neurite outgrowth on aggrecan in the presence of HB-GAM. Phospholipase C was found to abolish the neurite outgrowth-promoting effect of HB-GAM on aggrecan (Fig. 3d,i). Furthermore, downregulation of glypican-2 expression in CNS neurons (Supplementary Fig. S5) clearly inhibited the neurite outgrowth-promoting effect of HB-GAM on aggrecan (Fig. 3e,f,j). Altogether, the data showed that glypican-2 located on the neuron surface is required to mediate neurite outgrowth on aggrecan in the presence of HB-GAM.

### HB-GAM inhibits PTPσ binding to substrate-bound CSPG

**Figure 4:**
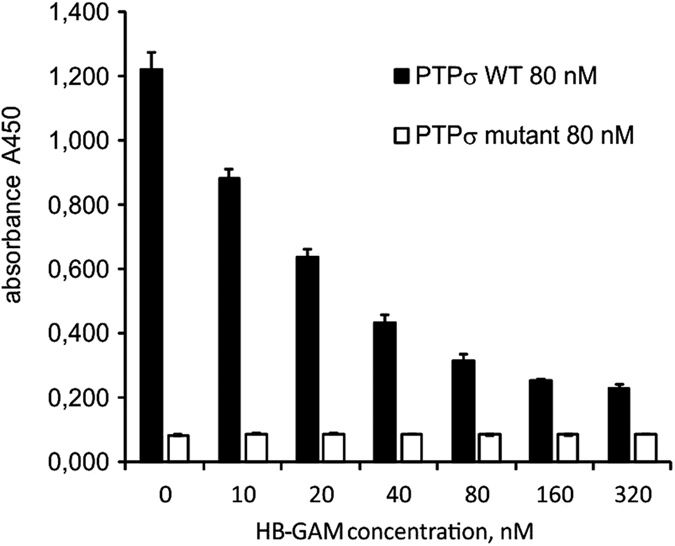
HB-GAM prevents PTPσ binding to chondroitin sulfates of aggrecan. Extracellular domain of wild type and mutant PTPσ (80 nM) was mixed with different concentrations of HB-GAM. The mutant PTPσ lacks the CS binding site. The mixture was added to wells coated with aggrecan (10 μg/ml) and bound PTPσ was measured with HPR conjugated goat anti-human IgG (FC part) using the O-phenylendiamine dihydrochloride colorimetric assay.

The transmembrane tyrosine phosphatase PTPsigma (PTPσ) regulates neurite outgrowth and acts as a nexus for multiple protein and proteoglycan interactions. It was recently shown to mediate CSPG inhibition of neurite outgrowth through binding to the CS side chains of CSPGs^19^,^20^,^21^. Since HB-GAM has strong binding affinity to the CS and HS chains of proteoglycans, we hypothesized that HB-GAM might compete with PTPσ for CS binding thus reducing the CSPG inhibition. We devised solid phase binding assays mimicking conditions of the neurite outgrowth experiments using PTPσ ectodomain and a PTPσ mutant lacking the CS binding site^19^. The mutant PTPσ displayed little if any binding to substrate-bound aggrecan compared to the wild-type PTPσ. Binding of the wild-type PTPσ to substrate-bound aggrecan was dose-dependently inhibited by HB-GAM (Fig. 4). Furthermore, we found that HB-GAM displayed binding to CS but not to PTPσ in a solid phase binding assay showing that HB-GAM binds to aggrecan and not to PTPσ(Supplementary Fig. S4f), and PTPσ was not found as an HB-GAM-binding component in bead assays using CNS neurons (Table 1). Altogether, the data revealed a competition between HB-GAM and PTPσ for the CS chains on aggrecan.

PTPσ is reciprocally regulated by interactions with CS or HS containing extracellular proteoglycans in a mechanism called the proteoglycan switch^20^. When ligated by CS chains PTPσ inhibits neurite outgrowth whereas it enhances neurite growth when ligated by HS chains. HS glycosaminoglycans, such as those on glypican-2, form clusters with PTPσ, a characteristic proposed to drive a localized imbalance of protein tyrosine phosphorylation and hence promotion of neurite outgrowth^20^. We therefore considered whether HB-GAM would shift PTPσ function from inhibition to outgrowth promotion. This however was not the case as knockdown of PTPσ in cortical neurons did not change neurite outgrowth on CSPG in the presence or absence of HB-GAM (Supplementary Fig. S4g–k).

### HB-GAM targets the glial scar region and promotes dendritic regeneration in the cerebral cortex

Since we found that HB-GAM was able to reverse the CSPG inhibition of neurite extension in brain neurons *in vitro*, we explored whether HB-GAM might also enhance dendritic and/or axonal regeneration *in vivo*. To explore the ability of HB-GAM in promoting dendritic regeneration *in vivo*, we used a prick-injury model of the cerebral cortex^22^. Dendritic regeneration was accurately followed in the injured cortical grey matter by *in vivo* two-photon microscopy through a chronic glass window implanted over the exposed cortex of adult transgenic mice with a subset of neurons (about 60% of layer V pyramidal cells) expressing yellow fluorescent protein^23^.

**Figure 5:**
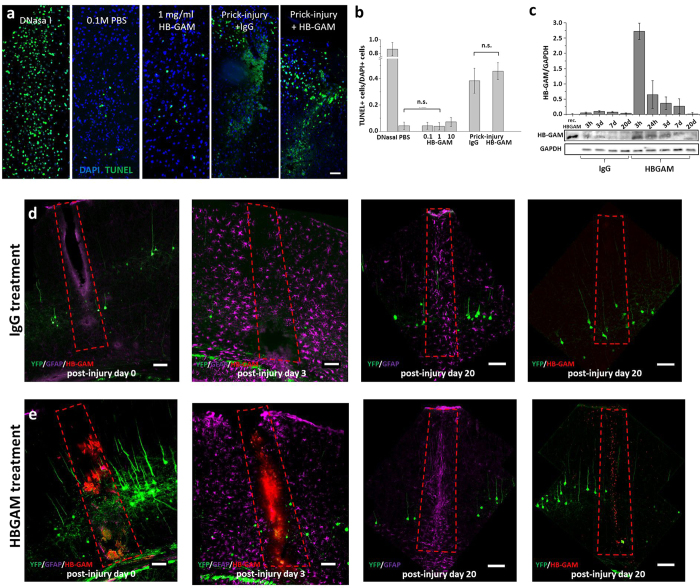
Lack of toxicity, time course of disappearance and distribution of injected HB-GAM in adult mouse neocortex. (**a**) Cortical sections from adult C57Bl mice were processed for TUNEL staining (green) to mark apoptotic cells (note – not specific for neurons) at day 3 post-injection or 3 days after the prick-injury followed by microinjection of IgG or HB-GAM. Brain sections were treated with DNase I for positive control. DAPI (blue) was used to label cell nuclei. Scale bar, 30 μm. (**b**) The fraction of TUNEL^+^ cells was calculated (mean ± SEM) to quantify apoptosis following the microinjection of different HB-GAM concentrations (0.1, 1.0 and 10.0 mg/ml). There was no significant difference in the numbers of TUNEL^+^ cells between animals injected with PBS, IgG or with different concentrations of HB-GAM. In addition, quantification of TUNEL staining did not show a significant difference in HB-GAM-injected animals after prick-injury compared to IgG-injected animals (1.5 μl of 1.0 mg/ml either HB-GAM or IgG). (**c**) Western blotting showing HB-GAM protein levels in somatosensory cortex lysates from the injury site following IgG or HB-GAM microinjection. GAPDH was used for normalization. Error bars represent SEM. (**d**) Coronal sections from Thy1-YFP mouse brains (neurons - green) 3 hours, 3 and 20 days after the injury following IgG treatment (1.5 μl at 1 mg/ml). Microphotography of GFAP (magenta) and HB-GAM (red) immunoreactivity shows that astrocytes are activated in a substantial volume of the brain cortex 3 days after the injury. Astrocytic scarring is seen in the injury site on post-injury day 20, but immunohistochemistry did not detect injury-induced HB-GAM expression. (**e**) Coronal sections from Thy1-YFP mouse brains 20 days after the injury following HB-GAM injection (1.5 μl at 1 mg/ml). As in control animals in (**d**), GFAP immunoreactivity shows astrocyte activation and astrocytic scarring. Notice the HB-GAM distribution in the injury site on post-injury day 20. The scale bar in (**a**,**d**,**e**) is 100 μm.

Different concentrations (0.1, 1.0 and 10 mg/ml) of baculovirus-derived recombinant HB-GAM^24^ were microinjected (1.5 μl) in normal and injured cortex. We did not observe any effects on cell survival resulting from the treatment either in the non-injured or in the prick-injured cortex when assessed by TUNEL staining (Fig. 5a,b).

HB-GAM injected into the site of prick-injury was found to accumulate to the area of astrocytes forming the inhibitory glial scar where activated astrocytes produce a CSPG-rich scar that inhibits regeneration and plasticity^1^,^3^,^4^,^7^. Interestingly, injected HB-GAM was detected by western blotting as a non-fragmented protein at least until day 7 (Fig. 5c) and by immunohistochemistry at the site of injury at least until day 20 after the injection (Fig. 5d,e), in agreement with the long half-life observed previously for the matrix-bound HB-GAM^25^. In contrast to the HB-GAM-injected mice, no HB-GAM signal was detected at the site of injury after injection of control IgG on days 0, 3 or 20 (Fig. 5c,d), demonstrating that the detected HB-GAM was the injected protein and did not result from an endogenous expression of HB-GAM induced by injury. Western blotting was not sensitive enough to detect HB-GAM on day 20 after the injury, likely because the remaining HB-GAM present at day 20 is restricted to the area of the astrocytic scar (Fig. 5e) and diluted in crude tissue lysates.

**Figure 6:**
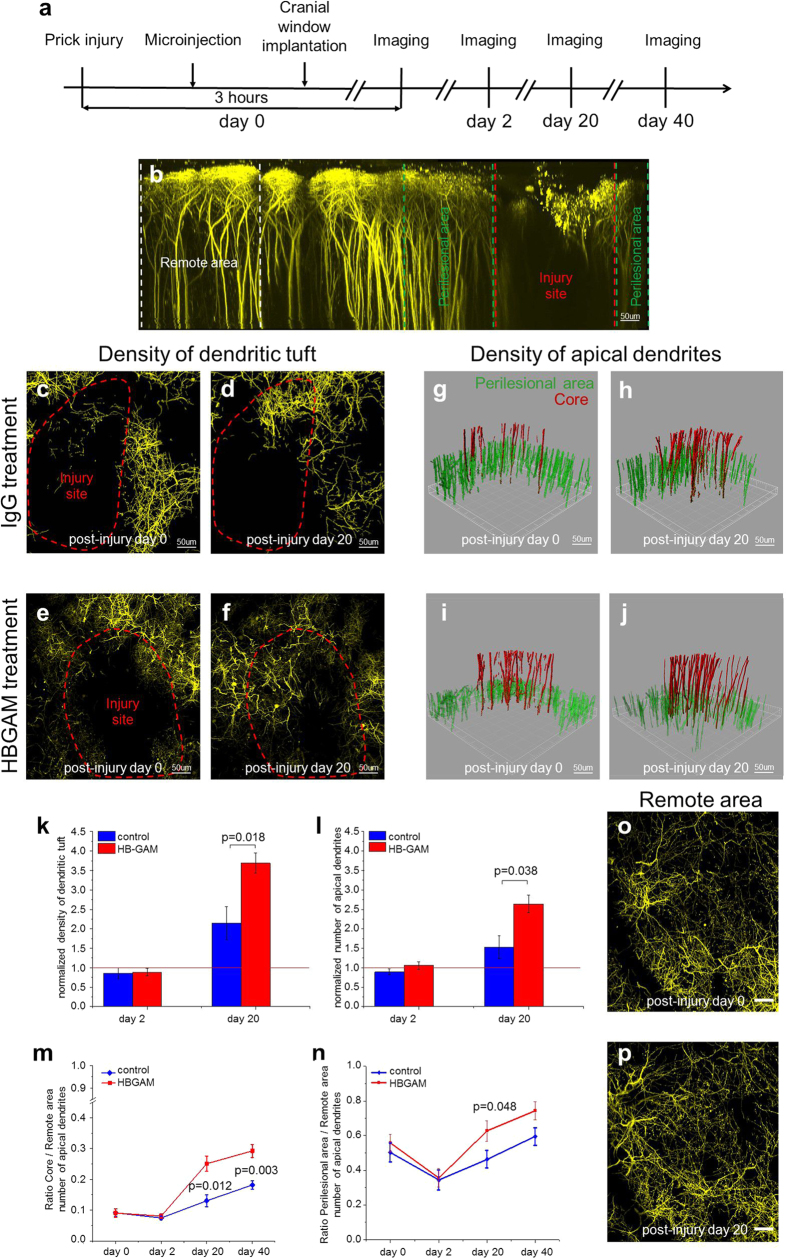
HB-GAM improves dendrite regeneration in injury site after prick-injury *in vivo*. (**a**) Experimental timeline. (**b**) The maximum projection of orthogonal reconstructions (side view) from a stack acquired by *in vivo* two-photon microscopy at 0 d after injury mark with dashed lines corresponding to the injury site (red), perilesional (green) and remote (white) areas. (**c**–**f**) The maximum projection (top view) of YFP-labelled cortical neurons dendritic tuft (yellow) 3 hours and 20 days after injury following IgG treatment in (**c**,**d**) or HB-GAM treatment in (**e**,**f**). Injury site (red dashed line) was defined from second harmonic signal in pia mater that indicates borders of injury. Notice the limited fraction of the dendritic tuft number regenerated in the injury site in response to IgG treatment in (**d**) compared to HB-GAM treatment in (**f**). (**g**–**j**) 3D reconstruction of YFP-labelled apical dendrites 3 hours and 20 days after injury following IgG treatment in (**g**,**h**) or HB-GAM treatment in (**i**,**j**). Apical dendrites in the injury core are color-coded in red and in the perilesion area in green. (**k**–**n**) Average density of dendritic tufts in (**k**) and average number of apical dendrites in (**l**) in injury site normalized to the time point of 3 h following control (blue column) and HB-GAM treatment (red column). Average ratio of the numbers of apical dendrites in the injury site in (**m**) and perilesional area in (**n**) compared to remote area over time in control experiments (blue line) and following HB-GAM treatment (red line) (p-value from Mann-Whitney-U test). Error bars, SEM (n = 6 control; n = 7 HB-GAM). (**o**) The maximum projection (top view) of dendritic tuft (yellow) in remote area 3 hours and (**p**) 20 days after injury.

Since the injected HB-GAM targets the astrocytic scar region, likely due to its avid binding to the dense deposition of the CS chains of CSPGs at the scar^3^,^4^,^7^, reversal of the CSPG inhibition might allow for dendritic regeneration in the injured cortex. In our prick-injury model we followed the HB-GAM effect on dendritic regeneration in the injury site, in the perilesional area, and in an area remote from the lesion site (experimental protocol summarized in Fig. 6a,b). *In vivo* two-photon microscopy revealed robust regeneration of the dendritic tuft and of apical dendrites compared to the start of the experiment (3 h from the acute injury) within 2–3 weeks in the core and perilesional area in HB-GAM-injected cortical injury sites compared to the controls (Fig. 6c–j; optical sections included in the merged image stacks shown in Video 1). Quantification of the experiment showed significant regeneration based on the density of the dendritic tuft (Fig. 6k) and on the number of apical dendrites normalized to the corresponding distant area of the cortex (Fig. 6l).

In a subset of experiments, we were able to follow the number of YFP+ layer 5 pyramidal neurons over time. The number of apical dendrites per neuron within the injury core and perilesional area was found to be increased in HB-GAM treated animals when normalized to the number of YFP+ neurons in the layer 5 (Supplementary Fig. S6). The dendritic tuft and the number of YFP+ neurons remained unchanged over time in remote area, supporting the conclusion that HB-GAM treatment promotes dendritic growth specifically at the lesion site and perilesional sites over time (Fig. 6m–p; Video 1).

### HB-GAM promotes axonal regeneration through spinal cord injury sites

**Figure 7:**
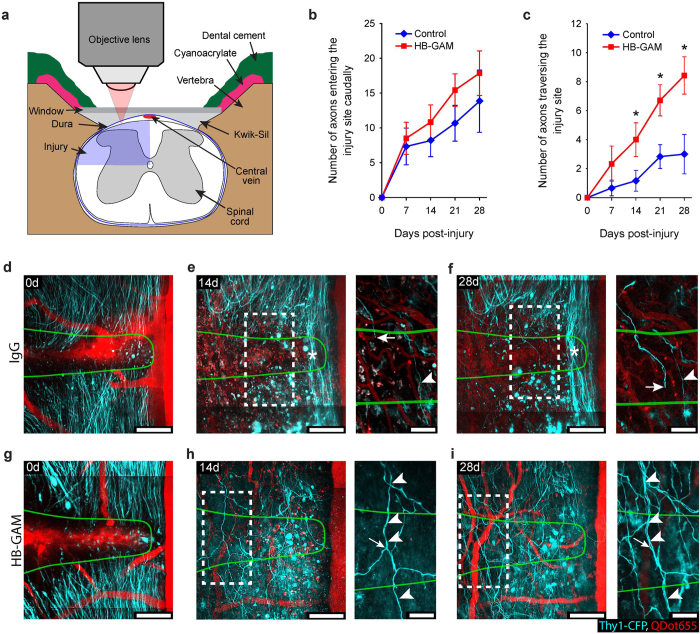
HB-GAM improves axon regeneration through transection injury sites in spinal cord *in vivo*. (**a**) Experimental set-up. (**b**,**c**) Average number of axon shafts at the caudal edge of the lesion site in (**b**) and average number of axons that had crossed the entire rostrocaudal extent of the injury site in **c** over the time following IgG and HB-GAM treatment (*p < 0.05; Mann-Whitney-U test). Error bars, SEM (n = 6, controls; n = 7, HB-GAM-treated mice). (**d**–**i**) Through-depth image stacks showing sprouting of cut dorsal column axons into and across spinal cord injury sites following IgG treatment in (**d**–**f**) or HB-GAM treatment in (**g**–**i**). Caudal is up, Rostral is down. Green lines show the outlines of the injury sites as defined at 0 days. Each image is the through-depth average of multiple optical-slice images ranging from 22–35 (44–70 μm) optical slices for main images, and from 6–9 (12–18 μm) optical slices for insets. (**e**,**f**,**h**,**i**) show the same regions as shown in (**d**,**g**) respectively, at 14 and 28 days after injury. Notice the limited number of axons crossing the rostrocaudal extent of the injury site in response to IgG treatment in (**e**,**f**) compared to HB-GAM treatment in (**h**,**i**). The stars in (**e**,**f**) show region containing spared axons. Insets in (**e**,**f**,**h**,**i**) show examples of individual axons that were identified at multiple post-injury times. The arrowhead in (**e**,**f**) indicates an axon that had crossed the spinal cord injury site at 14 and 28 days. The arrow in (**e**,**f**) indicates the distal tip of the same axon within the lesion site. The arrowheads in (**h**,**i**) point to an axon that had crossed the injury site at 14 and 28 days. The arrow in (**h**,**i**) points to an axon branch located within the injury site at 14 days, but was no longer detected at 28 days. Scale bars are 100 μm for main images and in 50 μm for insets.

CSPGs are also potent inhibitors of axonal regeneration and plasticity in the injured spinal cord^1^,^3^,^4^,^6^,^7^. To test whether HB-GAM could promote axon regeneration across spinal cord injury sites, adult Thy1-CFP mice (blue fluorescent axons in dorsal columns) were subjected to dorso-lateral spinal transection injuries (the experimental protocol summarized in Fig. 7a), immediately followed by injection of HB-GAM or IgG/vehicle (control experiments) at the injury site (5 μl at 1 mg/ml). Glass windows were implanted over the exposed spinal cords for repeated *in vivo* two-photon imaging of the injury sites^26^,^27^,^28^ from 0 to 4 weeks.

Since dorsal column Thy1-CFP axons typically project towards the brain (i.e., from caudal to rostral), the number of axons that entered the injury site along the caudal edge and the number of axons that crossed the entire rostro-caudal extent of the injury site were counted for each imaging session. Starting at 7 days the numbers of axons entering the injury site steadily increased for both control and HB-GAM treated spinal cords (Fig. 7b). The number of axons traversing the entire injury site also increased over time for both treatment groups. However, whereas the numbers of axons crossing the injury sites remained unchanged between 21 and 28 days for control animals, the numbers of axons crossing the injury sites of HB-GAM treated spinal cords continued to rise throughout the experiment and had significantly more axons that crossed the injury sites compared to the controls starting at 14 days post-injury (Fig. 7c). Consistent with our *in vitro* data, these *in vivo* imaging results support the idea that HB-GAM promotes axon growth after spinal cord injury, which can result in improved axon regeneration across the injury sites.

A closer view of axons at the injury sites revealed that immediately after window implantation there were no axons that crossed the injury sites of any of the control or HB-GAM treated spinal cords (Fig. 7d,g; optical sections included in the merged image stacks shown in Video 2). By 7 days and continuing to the final imaging sessions at 28 days both control and HB-GAM treated spinal cords had axons that traversed the entire rostrocaudal extent of the injury sites. For control spinal cords the axons within the lesion sites had simple morphologies with few branches and varicosities (Fig. 7e,f; optical slice images shown in Videos 3 and 4). Conversely, HB-GAM treated spinal cords had axons that were brightly fluorescent with numerous branch points and varicosities, and groups of axons often followed similar paths across injury sites (Fig. 7h,i; Videos 3 and 4). This is consistent with earlier observations^29^ that axons with complex growth terminals have a higher chance of successfully regenerating across injury sites than axons with simple growth terminals. Axons could be reliably observed in subsequent imaging sessions based on their morphologies and neuroanatomical markers (Fig. 7e,f,h,i, arrowheads). In general, once an axon had successfully regenerated across the lesion site it did not retract. In addition, when the growth and/or retraction of individual axon branches could be tracked at multiple time points we observed that axon growth and pruning continued for several weeks after spinal cord injury in both control and HB-GAM treated animals (for example, Fig. 7e,f for growth over time and Fig. 7h,i for retraction over time). In cases where an axon growth terminal could be identified in multiple imaging sessions (e.g., Fig. 7e,f) the rostral progression of the growing axon was measured over time. Consistent with our other data we found that HBGAM treated axons grew significantly faster (average = 23 ± 3 μm/day, n = 23 axons) than control axons (average = 9 ± 2 μm/day, n = 17 axons; p < 0.001; Mann-Whitney-U test). Also by tracking individual axons over time (Fig. 7h,i), we found that groups of crossing axons in HB-GAM treated spinal cords were formed by one axon regenerating across the injury site followed by other axons following similar trajectories at later times. These data show that early regenerators could act as ‘pioneer’ axons and provide guidance to axons that regenerate at later time points, and suggest that HB-GAM may facilitate these interactions between regenerating axons and axons that have already traversed the injury sites.

## Discussion

Treatments modulating the effects of glial scar on neurons have become a leading therapeutic approach in the field of CNS injuries^1^,^3^,^4^,^6^,^7^. It is generally accepted that the CS side chains of CSPGs are major inhibitors of neurite growth in the glial scar. In addition to their inhibitory role in the glial scar after injury, the CS chains are implicated in physiological regulation of CNS functions. For example, CSPGs accumulate in perineuronal nets of the healthy CNS at the end of the critical period of development and their CS side chains are reported to inhibit plasticity underlying behavioral regulation^2^,^5^,^11^. Therefore, the CS interactions with neurons are of wide biomedical interest, playing a role in the regulation of neural plasticity in the injured and non-injured CNS.

Digestion of the CS chains by chondroitinase ABC has been widely used to demonstrate the key role of the CSPGs in the regulation of regeneration and plasticity in the CNS^1^,^2^,^3^,^4^,^5^,^6^,^7^. However, CSPGs of the glial scar also have beneficial effects in the CNS following injury. For example, they are implicated in modulating immune responses and in regulation of progenitor cell proliferation^30^. We therefore sought for alternative ways to more specifically abrogate the neurite growth inhibition by the CS side chains of the CSPGs in the injured CNS.

HB-GAM is a candidate protein to modulate the effects of the CS chains on neurons since it binds avidly (at nanomolar K_D_ values) to the CS chains^12^ and lines nearly all fiber tracts in juvenile brain, but is strongly downregulated in the adult brain^10^,^15^. Our *in vitro* results show that CSPG matrices inhibit neurite outgrowth of CNS neurons, but HB-GAM added either in solution or coated on substrate with CSPGs abolishes the inhibitory effects of CSPGs and promotes robust neurite outgrowth. Importantly, HB-GAM can promote neurite outgrowth when added to cultures after they have already been inhibited by the CSPG.

**Figure 8:**
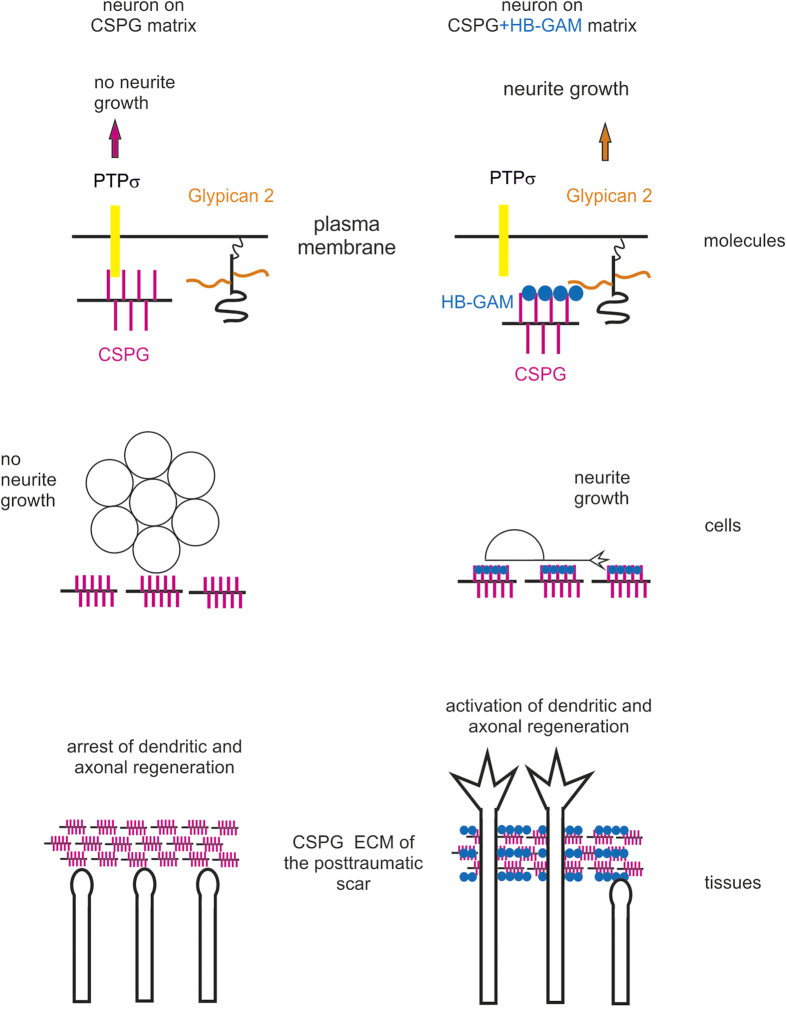
Summary of the current study. HB-GAM forms a complex with substrate-bound CSPGs and induces neurite outgrowth by binding to glypican-2 at the neuron surface. In addition, HB-GAM abrogates binding of PTPσ to CSPGs. At tissue level, CSPG-dependent inhibition of neurite outgrowth is seen as regeneration failure at the CSPG-rich astrocytic scar. As *in vitro*, inhibition of regeneration by the CSPG-rich matrix is reversed by HB-GAM as demonstrated by *in vivo* two-photon microscopy for dendritic regeneration in the cortex and axonal regeneration in the spinal cord.

In agreement with the generally held view, our findings show that chondroitinase ABC reduces CSPG inhibition of neurite outgrowth. However, chondroitinase ABC also abolishes the capability of soluble HB-GAM to induce neurite outgrowth on CSPG substrate. Therefore, HB-GAM reverses the effects of the CS chains from potent inhibitors to potent activators of neurite outgrowth (see the summary of the current study in Fig. 8). This finding agrees with our previous studies showing that HB-GAM in solution does not enhance neurite outgrowth but may even be growth inhibitory^24^. Collectively, these data suggest that CS chains may provide ideal structures to trap HB-GAM from solution to make a polyvalent interaction surface that enhances neurite growth (Fig. 8).

PTPσ has been identified as a CS receptor and a primary mediator of CSPG inhibition^19^,^20^,^21^. We show that HB-GAM is a potent inhibitor of PTPσ binding to CS chains. This inhibition is observed for wild-type PTPσ but not for mutant PTPσ lacking the CS binding site. Moreover, HB-GAM does not bind to PTPσ as shown by surface plasmon resonance assays in which HB-GAM binding to CS is observed as expected^12^. The inhibition of PTPσ binding to CS chains is therefore due to competition of HB-GAM and PTPσ for the CS binding sites. Competition for CS binding sites may therefore reduce PTPσ mediated inhibition of neurite outgrowth. However, this mechanism does not account for the growth promoting effects of HB-GAM on CSPG substrates since HB-GAM had similar effects on neurite outgrowth in PTPσ knockdown cultures.

PTPσ is reported to enhance or inhibit neurite growth depending on its mode of binding to glycosaminoglycan chains^20^. It is therefore possible that the CS-bound HB-GAM would switch the PTPσ signaling from a negative to a positive mode. However, this possibility is also unlikely given the finding that PTPσ knockdown has no effect on neurite outgrowth in the presence of HB-GAM.

We therefore sought for neuron surface receptors that would explain the neurite outgrowth effect of HB-GAM on CSPG substrates. We observed that heparinase III abrogates the effect, suggesting that HS chains of the neuron surface are required. Since syndecan-3 (N-syndecan) has previously been identified as the neuron surface receptor of HB-GAM^18^,^31^, one would expect that syndecan-3 acts as the heparinase III sensitive receptor mediating HB-GAM-induced neurite growth on CSPG substrates. However, this is not the case since both wild-type and syndecan-3 knockout neurons have similar neurite outgrowth on CSPG substrate in the presence of HB-GAM. Interestingly, unbiased search of HB-GAM binding sites in CNS neurons identified glypican-2 as a receptor candidate. Indeed, phospholipase C abrogated the HB-GAM-induced neurite outgrowth on CSPG substrate, which clearly suggests a role for a GPI-linked cell surface receptor such as glypican-2. Furthermore, downregulation of glypican-2 using siRNA inhibited the HB-GAM effect. These results strongly suggest that glypican-2 at the neuron surface is required for HB-GAM-induced neurite outgrowth on CSPG substrate. Overall, our results suggest that HB-GAM acts as a linker from matrix CS chains to neuron surface HS chains of glypican-2 (Fig. 8).

The finding of glypican-2 as a neurite outgrowth receptor in mammals is consistent with the findings that glypican-2 is highly expressed in mammalian central neurons that extend neurites^32^ and that its ortholog Dally-like in *Drosophila* plays a role in axon guidance^33^. Interestingly, glypican-2 has also been identified as a neurite outgrowth receptor of Midkine^34^. Developmental studies have previously shown that glypicans regulate signaling of Wnts, Hedgehogs, fibroblast growth factors and bone morphogenetic proteins^35^. Moreover, binding of HB-GAM to the GPI-anchored glypican-2 might also explain the unexpected enrichment of HB-GAM in lipid raft fractions observed previously in fractionation of brain tissue^31^. GPI anchors act as targeting motifs to lipid raft microdomains that are considered to be important platforms for signal transduction in neurons^36^. Further studies are warranted to elucidate molecular interactions of HB-GAM/glypican-2 in lipid rafts and downstream signaling during the regenerative response.

Our *in vivo* results using CNS trauma models support the *in vitro* analyses. Injection of HB-GAM into injured cerebral cortex demonstrates HB-GAM accumulation to the area of activated astrocytes that are known to produce CSPGs in the glial scar^1^,^3^,^4^,^7^. Furthermore, intravital two-photon microscopy reveals a robust regenerative response of injured cortical dendrites to HB-GAM. Similarly, recurrent two-photon imaging of sensory axons in the injured adult mouse spinal cord through implanted glass window^26^ reveals that HB-GAM injected into the lesion site significantly increases the number of axons traversing the injured area starting 14 days after the injury. Whereas this number appears to stabilize by 21 days in controls, it continues increasing until day 28 in HB-GAM treated mice. A likely explanation for our *in vivo* observations, supported by the experiments *in vitro*, is that locally administered HB-GAM promotes dendrite and axon extension as an extracellular matrix factor bound to CSPGs that normally accumulate at CNS injury sites. This interpretation is also consistent with the finding that matrix-bound HB-GAM has a long half-life in tissue^25^. Indeed, HB-GAM injected into brain tissue can be detected by immuhistochemistry at least until 20 days after the injury. Furthermore, our detection methods may not be sensitive enough to show HB-GAM in tissue at concentrations at which it still displays activity in neurite growth. Another issue to be considered is the pioneer effect suggested by imaging in spinal cord: the pioneers growing fast during the first 1–2 weeks through the trauma area in HB-GAM-injected animals are expected to help follower neurites to grow through the injury site even after the injected HB-GAM has disappeared.

The generally accepted concept that CSPG matrices inhibit regeneration and plasticity of the neural circuitry needs to be revised. We have now demonstrated that, through the use of HB-GAM, the CSPG matrix can be modulated to promote neural regeneration (summarized in Fig. 8). HB-GAM, and molecules displaying similar binding properties, may serve as leads for the development of therapeutic strategies that enhance neural regeneration following traumatic brain and spinal cord injuries that currently have extremely limited therapeutic options.

## Materials and Methods

### Reagents

HB-GAM was expressed in Sf 9 cells using a baculovirus vector and purified from the culture medium of the cells as described previously^24^. Aggrecan, IgG, chondroitin sulphate from shark cartilage, phospholipase C and heparinases I-III were purchased from Sigma. Chondroitinase ABC (Proteus vulgaris) was purchased from Amsbio. Neurocan was from Millipore. A mixture of soluble proteoglycans that contain mainly CSPGs^37^ was isolated from embryonic day 16–18 rat brains using anion exchange chromatography on DEAE-Sepharose. Proteoglycan fractions in NaCl gradient elution from DEAE-Sepharose were detected by uronic acid determination, pooled and treated with heparinase III to remove the HS chains of the samples. The N-terminal extracellular part of PTPσ that contains the three Ig domains (amino acids 1–321) and mediates CSPG binding^19^ was cloned using the PTPσ cDNA kindly provided by Dr. John Flanagan (Harvard University). The PTPσ mutant lacking the CS binding site in the Ig1 domain was produced as described^19^. The recombinants were produced as Fc-tagged proteins in 293T HEK cells and purified on protein A Sepharose. PTPσ morpholino was from Gene Tools (Oregon). Affinity-purified polyclonal antibodies against the recombinant rat HB-GAM^24^ and polyclonal antibodies against human pleiotrophin (Abcam) were used to detect HB-GAM. Anti-GFAP antibodies (Abcam) were used to detect the glial scar.

### Cells and cell culture

Hippocampal and cortical cell cultures were prepared from E17 Wistar rat embryos or from P0-P4 C57BL/6JOlaHsd mice. Cells were cultured in 48-well plates (Nunc) at the density 5 × 10^4 ^cells/well or 96-well plates (Nunc) at the density 10^4^ cells/well in Neurobasal medium supplemented with B27 serum substitute, penicillin, streptomycin and L-glutamate. Plate coating with CSPGs, HB-GAM or IgG was performed overnight at +37 °C. Where indicated, aggrecan-coated substrate was treated with chondroitinase ABC (2 U/ml) for 30 min before plating cells. Phospholipase C (0.4 U/ml) was added to cells 1 h before plating cells on the aggrecan-coated substrate. Heparinases I-III were added when plating cells. PC12 cells (PC6 subclone) were cultured in DMEM supplemented with 10% fetal calf serum, penicillin and streptomycin. All experimental protocols for neuronal cell culture experiments of the study were approved by ELLA- Animal Experiment Board in Finland (the permission numbers: ESLH-2008-09065/YM-23 and ESAVI/11326/04.10.07/2014). The methods for neuronal cell culture experiments were carried out in accordance with the guidelines of the Animal Experiment Board in Finland.

### Immunocytochemistry and morphometry

Neuronal cultures were fixed and stained with mouse monoclonal antibody against tubulin β III (Millipore, catalog number MAB1637). Alexa-568-conjugated goat anti-rabbit secondary antibodies were from Molecular Probes. In some experiments, live or PFA-fixed cultures were imaged using phase contrast microscopy. All cell cultures were imaged using the x 20 objective (numerical aperture 0.40) of the Olympus IX70 microscope connected to Retiga 2000R digital camera (QImaging). Neurite length was measured with ImagePro Plus and ImageJ software. In all *in vitro* experiments statistical significance was calculated for the data from at least 3 independent experiments using one-way ANOVA (Excel, Microsoft). Error bars represent standard error of mean (SEM), symbols *, ** and *** represent P < 0.05, 0.01 and 0.001, respectively. Kolmogorov-Smirnov test was used to verify normality of distribution. Image acquisition was non-blind, image analysis (neurite length measurement) was performed in a blind manner.

### Solid phase binding assays

Nunclon delta 96-well tissue culture plates (Nunc) were coated overnight at 4 °C with 10 μg/ml aggrecan in 1 × PBS. The wells were washed two times briefly with 0.05% Tween-20 in PBS and were blocked for 1 h at room temperature with 1%BSA and 0.05% Tween-20 in PBS. The PTPσ WT and the PTPσ mutant lacking the CS binding site were Fc fusion proteins. The PTPσ recombinants (80 nM) were mixed with different concentrations of recombinant HB-GAM in 1% BSA and 0.05% Tween-20 in PBS, and the mixture was incubated in the aggrecan-coated wells overnight at 4 °C. The wells were washed three times with 0.05% Tween-20 in PBS at room temperature. Bound PTPσ was detected by incubating the wells for 1 h at room temperature with HRP conjugated goat anti-human IgG (FC part) which was diluted 1/10 000 in 1% BSA and 0.05% Tween-20 PBS. The wells were then washed three times with 0.05% Tween-20 PBS. The enzymatic activity of the bound HRP conjugate was measured at 450 nm by using O-phenylendiamine dihydrochloride (OPD, SIGMA P9187).

Surface plasmon resonance studies were conducted with a Biacore T100 instrument (GE healthcare). HB-GAM was immobilized to a CM5-chip (GE healthcare), and a blank channel was used as the reference. Samples were injected at 20 μl/min flow rate with 60 s injection times. Shark CS was injected at 300 nM and PTPσ at 1 μM and 5 μM concentrations.

### Search for HB-GAM receptors using pull-down assay and mass spectrometry

HB-GAM and BSA (as a control) were covalently bound to long-arm epoxy-activated magnetic beads BcMag™ (Bioclone). The coupling was done at 4 °C, pH 9.5, during 48–72 h. After 6 washing steps with PBS buffer, the beads were resuspended in PBS (10 mg/ml).

Hippocampal primary neurons were cultured in Neurobasal media for 3–7 days in 6-well plates. The cells were washed twice by PBS buffer, the beads were added to the neurons and incubated for 2 h at 4 °C with gentle shaking followed by 30 min incubation with 2 mM concentration of the cross-linking reagent DTSSP (Pierce).

After lysis of the cells by RIPA buffer with 50–100 mM n-dodecyl β-D-maltoside (Life Technologies), a transmembrane fraction coupled to the beads was collected by magnets, and the LC-MS analysis was performed on an Orbitrap Elite ETD mass spectrometer (Thermo Scientific) using the Xcalibur version 2.7.1 coupled to a Thermo Scientific nLCII nanoflow system (Thermo Scientific) *via* a nanoelectrospray ion source. Peak extraction and subsequent protein identification were achieved using Proteome Discoverer software (Thermo Scientific). Calibrated peak files were searched against the Rattus norvegicus component of UniProtKB/SwissProt database (http://www.uniprot.org) by a SEQUEST search engine.

The total number of hits was 334 in two independent samples from hippocampal primary neurons (E17–18). After subtraction of hits which were common with BSA controls, a list of potential interacting proteins was sorted by SEQUEST probability based scoring.

### Glypican-2 siRNA treatment of neurons

Rat primary hippocampal neurons were electroporated following instructions provided with Amaxa Rat Neuron Nucleofector Kit (cat. no. VPG-1003). Briefly, 5 × 10^6^ hippocampal neurons were electroporated/nucleofected with the mixture of three rat glypican-2 specific siRNAs (Qiagen cat. no. SI00282513, SI03039568 and SI03094770). For one treatment 0.5 μl of each siRNA (20 μM) was added. As controls, the neurons were treated with 1.5 μl of negative control siRNA (20 μM, Qiagen cat. no. 1022076). Immediately after the electroporation 60 × 10^3^ rat hippocampal neurons were plated on Neutravidin wells to which biotinylated aggrecan had been linked (96-well format, Thermo Scientific cat. no. 15128) with or without HB-GAM in solution (20 μg/ml). Total neurite length per field was measured after 2 DIV.

To detect the efficiency of the glypican-2 siRNAs in reducing glypican-2 expression, 2 × 10^6^ electroporated hippocampal neurons per treatment were plated on 6-well plates coated with poly-L-lysine. After 2 DIV both the glypican-2 siRNA and negative control siRNA neurons were treated for 2 h with heparinase III (IBEX) and were directly lysed by adding 100 μl Laemmli sample buffer per well. The samples were western blotted with goat anti-glypican-2 antibodies (SCBT, cat. no. sc-33992) with 1/500 dilution in soya milk, and for loading control purpose the same samples were blotted with mouse anti-GAPDH (SCBT, cat. no. sc-32233) with 1/500 dilution in 5% milk in PBS solution.

### Animal models

Transgenic mice with fluorescent dendrites and axons were used to study regeneration after acute brain prick injury and spinal cord injury with the aid of two-photon microscopy. Thy1-YFP mice^38^ were used to study dendritic regeneration in cortex, and Thy1-CFP-23 mice^38^ were used to study axonal regeneration in spinal cord. Syndecan-3 knockout mice^39^ were used to study the role of syndecan-3 (N-syndecan) in regeneration.

The mice were group-housed under standard conditions (food and water were available ad libitum) with 12 h/12 h lights on-off time (lights on at 6 p.m.), relative humidity 50–60%, and room temperature 21+/−2 1 uC. Mice from each cage were randomly distributed to all treatment groups. Animals with implanted windows were housed in individual cages after surgery.

All experimental protocols for surgical procedures for imaging of spinal cord injuries were approved by the National Animal Studies Committee of France (authorization no. 13,300) and by the National Committee for Ethic in Animal Experimentation (Section N°14; project 86-04122012). Methods for the spinal cord imaging were carried out in accordance with the guidelines of the National Animal Studies Committee of France. All experimental protocols for all other animal experiments of the study were approved by ELLA- Animal Experiment Board in Finland (the permission numbers: ESLH-2008-09065/YM-23 and ESAVI/11326/04.10.07/2014). The methods for animal experiments were carried out in accordance with the guidelines of the Animal Experiment Board in Finland.

### *In vivo* two-photon microscopy of somatosensory cortex after brain prick injury

To study changes in dendritic morphology after the brain prick injury, thirteen 2-month old Thy1-YFP mice were used. The mice were anaesthetized (i.p.) with a mixture of ketamine (80 mg/kg) and xylazine (10 mg/kg), and maintained at 37.0 °C using a heating pad during surgical operations and imaging sessions. The surgical procedures for producing cortical prick injury and preparing a chronic cranial window for *in vivo* two-photon imaging methods have been described previously^22^.

For repetitive *in vivo* microscopy^40^ (raw data of the optical sections shown in Video 1) the animal was placed under the FV1000MPE two-photon microscope (Olympus) by attaching the metal holder to the custom built frame. Two-photon excitation was achieved with MaiTai femtosecond laser tuned to 860–950 nm to excite YFP. Second harmonic signal in pia mater was used to define the injury site and borders of the prick injury. Using the 25X water immersion objective, in every experiment three imaging fields –injury site with perilesional area and two remote regions - were imaged 3 h after the cortical prick injury and followed at 48 h, 20 days and 40 days. Each imaging field comprised 500 × 500 μm in xy axes with a pixel size of 0.49 μm. Image stacks were collected with a vertical step of 2–3 μm. Most images of neuronal morphology could be visualized with the depth of penetration up to 800 μm below the cortical surface. Manual analysis of images was performed using ImageJ tools and Imaris software (Bitplane). Prior to analysis of the density of dendritic tufts and the number of apical dendrites, a surface was generated with Imaris software (number of voxels above 500 and sphericity below 0.75) to get rid of the blebbed dendrites and unspecific speckles. Everything outside the surface was erased, so only objects corresponding to neuronal morphology remained. The maximal intensity z-projection of YFP-labelled dendritic tuft of cortical neurons was taken within the first 120–180 μm of cortex (40–60 optical sections). To reduce noise from images a median filter with 1 pixel radius was applied to images. Further, images were converted from grayscale to binary and automatically segmented with Otsu’s thresholding method. The density of dendritic tufts was calculated as a sum of pixels belonging to dendrites per area of injury site, perilesional area or a remote region. For measurement of the number of apical dendrites, brightness and contrast of individual z-slices was normalized in Imaris to a uniform level. Next, we selected and aligned between 7 and 15 sequential sub-stacks (250 × 250 × 30 μm) below the first dendrite branch point of layer V neurons, typically deeper than 120 μm from the cortical surface. Apical dendrites were identified within each sub-stack using the Imaris ‘surfaces’ tool as objects above 100 voxels with ellipsoid axis length longer than 10 μm. The number of apical dendrites in an individual animal was calculated as an average normalized number of surfaces. All sub-stacks were normalized to the number of the apical dendrites at the 3 h time point. The N values refer to the number of independent replications, each of them performed in a different animal. Data were presented as mean ± SEM. For statistical analyses using Mann-Whitney-U test, the P < 0.05 value was considered significant.

### Fluorescent immunostaining and TUNEL assay for apoptosis after brain prick injury

For immunostaining experiments, 24 Thy1-YFP mice were used. The animals were deeply anaesthetized with CO_2_ (95%CO_2_, 5% O_2_) at 3 h, and at 3, 7 and 20 days after the prick injury. Fixation was done by transcardial perfusion with chilled PBS and 4% paraformaldehyde (PFA). The brains were then postfixed in 4% PFA overnight at +4 °C. After sinking in 30% sucrose, the brains were enabled in Tissue-Tek compound (Sakura), frozen at −80 °C and 25 μm thick coronal sections through the injury and control sites were cut with CM3050S cryostat (Leica). The slices were collected and stored at −20 °C in cryoprotective solution (30% sucrose, 1% polyvinyl-pyrrolidone (PVP-40), 30% ethylene glycol in PBS). Triple staining was done on floating sections. To prevent unspecific binding of the antibodies and streptavidin-biotin, the sections were incubated for 30–60 min with 5% normal donkey serum (Millipore), 5% goat serum (Gibco) and 3% BSA (Sigma) in PBS containing 0,5% Triton-X100 and then with streptavidin-biotin solutions (Vector laboratories). Affinity-purified rabbit polyclonal antibodies against recombinant HB-GAM (0.74 μg/ml), rabbit polyclonal antibodies against human pleiotrophin (1:200; Abcam) and chicken polyclonal antibodies against GFAP (1:2000; Abcam) were used as the primary antibodies. The primary antibodies were detected with Alexa 350 donkey anti-rabbit IgG and Alexa 546 goat anti-chicken IgG (1:10000; Invitrogen). The sections were mounted on slides and covered with anti-fade reagent (Fluoromount, Sigma). The control HB-GAM staining on E18 and P0 brains and the control without the primary antibodies was included in every immunostaining experiment.

For TUNEL assay for apoptosis a commercially available kit (Dead End Fluorometric TUNEL System, Promega) was used. Staining was performed according to supplier’s recommendations on the 7 μm coronal sections from paraffin-embedded brains. Positive control sections treated with DNase I were included on every slide. To visualize all nuclei, sections were counterstained with DAPI.

The images were taken with Axio Scop Rel. program in 16-bit format using the Imager M.1 fluorescent microscope (Zeiss). At the minimum 5 sections per hemisphere per animal were studied. Image processing was done with ImageJ software.

### Western blotting after brain prick injury

The brain samples were collected from 24 C57bl/6JRcc mice. The animals were anaesthetized (i.p.) with a mixture of ketamine (80 mg/kg) and xylazine (10 mg/kg) at 3 h, and 3,7 and 20 days after the pin-prick injury. The skull was drilled over the injury site to form a square with 2–2.5 mm sides, and the cranial bone was then carefully removed. Blood from cranial vasculature was washed by transcardial perfusion with cold PBS. A piece from the cortex was then cut by a 15° tab knife and homogenized in cold RIPA buffer (Sigma). After at least 15-minute incubation on ice, the samples were centrifuged (16000 g, 15 min, +4 °C) and the supernatants were collected for further analysis. Protein concentrations were measured using the BCA protein assay kit (Thermo Scientific). The samples (50 μg protein) and the recombinant HB-GAM (0.15 μg) as the positive control were separated on SDS-PAGE and blotted to a nitrocellulose membrane (300 mA, 1 hour, 4 °C). The membranes were incubated for 60 min with 3% bovine serum albumin (Sigma) in Tris-buffered saline containing 0.1% Tween (TBST). The membranes were incubated with the following primary antibodies: anti-recombinant HB-GAM^23^ (0.1 μg/ml) and anti-GAPDH (sc-25778; 1:4000; SCB). The membranes were then washed with TBST and incubated with secondary antibodies conjugated to horseradish peroxidase (Bio-Rad; 1:10000 in non-fat dry milk, 1 h at room temperature). Secondary antibodies were visualized using enhanced chemiluminescence (ECL Plus, ThermoScientific) and detected by LAS-3000 camera (FujiFilm). Densitometry was performed using ImageJ.

### Spinal cord surgery and glass window implantation

A total of 13 adult Thy1-CFP23 mice^38^ backcrossed to C57/Bl6 mice (>8 week old and >21.3 g) were used. The window implantation protocol has previously been described in detail^26^,^27^,^28^. Briefly, the T11 to L1 vertebrae were surgically exposed and a window support structure was implanted using the T11 and L1 vertebrae as anchoring points. A laminectomy was done on T12 and T13 vertebrae to expose the lumbar enlargement^40^,^41^. Using fine angled Vannas spring scissors (Fine Science Tools) the dorsallateral spinal cord was transected from the lateral dura opening to the spinal cord midline and approximately 0.5 mm deep from the dorsal surface of the spinal midline. Special care was taken to not cut the dorsal vein. Injuries were made roughly in the middle of the exposed spinal cord rostrocaudally (~L3 spinal segment). The mediolateral completeness of the lesions was verified using the two-photon microscope. Following the injury, 5 μl of either PBS (n = 2), IgG (n = 4; 1 mg/ml), or HB-GAM (n = 7; 1 mg/ml) was injected directly into the injury site through the dura opening for 10 minutes. We observed no differences between the PBS and IgG treatment groups, which are therefore simply called the IgG treated or control group. Immediately prior to implantation, the glass window was cleaned and dried, and the dura mater was rinsed with PBS, cleared of any debris, and allowed to dry until tacky. A line of liquid KwikSil (World Precision Instruments) was applied to the dura mater surface along the midline of the spinal cord, and the glass window was immediately placed over the spinal cord. The window was held in place using cyanoacrylate, followed by a layer of dental cement along the edges. Postoperative analgesia and prolonged window clarity were obtained by administration of dexamethasone (0.2 mg/kg) and rimadyl (5 mg/kg) s.c. every 2 days for 10 days after surgery.

### *In vivo* two-photon microscopy after spinal cord injury

The imaging protocol has previously been described in detail^26^,^27^,^28^. The exposed spinal cords were imaged immediately after window implantation and once every week. All animals were imaged for at least 4 weeks. Each imaging session lasted roughly one hour. For each imaging session mice were lightly anaesthetized with ~1.75% isoflurane (Baxter) (v/v) in air for ~2 min, followed by Ketamine/Xylazine (100 mg/kg; 10 mg/kg). Prior to each imaging session, 30 μl of QDot655 (Qtracker 655 non-targeted quantum dots; Invitrogen) (50 μl/ml in PBS) was injected i.v. Throughout the imaging sessions the animals were freely breathing and the microscope chamber was warmed to ~32 °C. Following each imaging session the animals were returned to their cage with a piece of tissue for nesting and kept warm until they recovered from anesthesia.

A tunable femtosecond pulsed laser (MaiTai, SpectraPhysics) was coupled to a Zeiss two-photon microscope (LSM 7 MP) equipped with a 20× water immersion objective lens (NA = 1.0) and five non-descanned detectors. The laser was tuned to either 840 nm or 940 nm, and filter sets were designed to optimize the separation of the emission spectra of the fluorophores. For each image stack laser intensity, and sometimes master gain, were adjusted according to imaging depth in order to maximize signal intensity while minimizing saturation throughout the image stack.

Images were analyzed using ImageJ software. Occasionally there was drift in the field of view within an image stack; these drifts were realigned using the StackReg plugin on ImageJ. Analysis was performed on registered image stacks, but for clarity all merged image stacks shown in figures were brightness and contrast enhanced. In cases where more than 10 optical sections were merged to form a single image, these merged images were filtered using the Unsharp Mask of ImageJ (Radius, 2.0 pixels; weight, 0.7). For all merged image stacks subjected to Unsharp Mask filtering, each of the optical sections included in the merged image is shown in through-depth videos (Videos 2–4).

Within each image stack we counted all stained axons that entered the lesion site across the caudal edge and those that traversed the entire rostrocaudal extent of the lesion. To start, using the initial imaging session on day 0 as a template, we outlined the lesion site as all regions containing either no CFP+ axons, CFP+ axon debris, and/or CFP+ retracting axons, and marked the location of the outline relative to distinct anatomical marker such as large uninjured blood vessels. The rostrocaudal center of the lesion was defined as a mediolateral line from the lateral edge of the image to the most medial extent of the lesion with roughly half the lesion site located rostral of the centerline and roughly half the lesion site located below the centerline. To determine the number of axons that entered the lesion, the number of axon shafts that crossed the centerline was counted throughout the entire depth of the image. To determine the number of axons that successfully regenerated across the entire rostrocaudal extent of the lesion, we first searched for an axon shaft that crossed the rostral edge of the lesion site. The axon was then followed caudally through the lesion site until it terminated, was no longer discernible, or crossed the caudal edge of the lesion site. Only axons that could be traced from the rostral to the caudal edge of the lesion were counted. This process was repeated for all axon shafts that crossed the rostral edge of the lesion site. For each subsequent imaging session, the lesion outlines were realigned to the same position using blood vessels as anatomical markers.

In some mice axons were observed near the central vein within one week after injury. These axons were numerous, arranged in parallel groups, had normal axon morphology, and were observed in both control and HB-GAM treated mice. We considered these axons as spared and did not include them in our quantitative analysis.

In addition to analyzing the number of axons that grew through the injury site, we calculated the growth rates of actively growing axons (more than 1 μm/day). In this analysis the rostro-caudal growth rates of individual axons through the injury sites were calculated for axons that could be identified in repeated imaging sessions. For this we searched the injury site of each image stack for axon growth terminals. To be considered an axon growth terminal the axon must be visible caudal of the lesion site and come to a termination (usually with a swelling) within or rostral of the lesion site. Once an axon growth terminal was identified in an image stack, we searched for the same axon in subsequent imaging sessions. The same axon was re-identified in multiple imaging sessions using anatomical landmarks (e.g., axon location in relation to the lesion sites, blood vessels, and/or other nearby axons) and morphological features of the re-identified axon (e.g., axon trajectory, branching, and axon diameter). Only axons that were located in the same place and had very similar morphological features across imaging session were considered as re-identified. Once an axon had been re-identified, the rostrocaudal location of the terminal of the axon was measured relative to other anatomical landmarks (usually injury border and/or blood vessels). The growth rate of each axon was then calculated by dividing the distance grown (μm) by the time between imaging sessions (days).

## Additional Information

**How to cite this article**: Paveliev, M. *et al.* HB-GAM (pleiotrophin) reverses inhibition of neural regeneration by the CNS extracellular matrix. *Sci. Rep.* **6**, 33916; doi: 10.1038/srep33916 (2016).

## Supplementary Material

Supplementary Information

Supplementary Video 1

Supplementary Video 2

Supplementary Video 3

Supplementary Video 4

## Figures and Tables

**Table 1:** Hits with the highest scores (SEQUEST) in mass spectrometry analyses of proteins from pull-down assay with HB-GAM and BSA magnetic beads from hippocampal neuronal cultures.

Accession	Description	Coverage BSA	#Peptides BSA	Score HBGAM_1	#Peptides HBGAM_1	Score HBGAM_2	#Peptides HBGAM_2
P63090	Pleiotrophin OS = Rattus norvegicus GN = Ptn PE = 1 SV = 1 - [PTN_RAT]			60.48	7	57.62	8
P11442	Clathrin heavy chain 1 OS = Rattus norvegicus GN = Cltc PE = 1 SV = 3 - [CLH_RAT]	0		42.81	10	50.11	15
P50137	Transketolase OS = Rattus norvegicus GN = Tkt PE = 1 SV = 1 - [TKT_RAT]	0		24.04	7	27.27	4
Q9EPH8	Polyadenylate-binding protein 1 OS = Rattus norvegicus GN = Pabpc1 PE = 2 SV = 1 - [PABP1_RAT]	0		17.45	5	23.63	6
P85845	Fascin OS = Rattus norvegicus GN = Fscn1 PE = 1 SV = 2 - [FSCN1_RAT]	0		15.33	3	13.67	3
P09330	Ribose-phosphate pyrophosphokinase 2 OS = Rattus norvegicus GN = Prps2 PE = 1 SV = 3 - [PRPS2_RAT]			13.82	3	10.67	2
Q4FZT9	26S proteasome non-ATPase regulatory subunit 2 OS = Rattus norvegicus GN = Psmd2 PE = 2 SV = 1 - [PSMD2_RAT]	0		13.44	2	3.14	1
P51653	Glypican-2 OS = Rattus norvegicus GN = Gpc2 PE = 1 SV = 1 - [GPC2_RAT]			12.36	3	9.39	3
Q9JMJ4	Pre-mRNA-processing factor 19 OS = Rattus norvegicus GN = Prpf19 PE = 2 SV = 2 - [PRP19_RAT]	0		12.14	3	20.66	7
Q63610	Tropomyosin alpha-3 chain OS = Rattus norvegicus GN = Tpm3 PE = 1 SV = 2 - [TPM3_RAT]	0		11.69	5	12.79	5
O88761	26S proteasome non-ATPase regulatory subunit 1 OS = Rattus norvegicus GN = Psmd1 PE = 2 SV = 1 - [PSMD1_RAT]	0		10.28	2	10.48	3
P61765	Syntaxin-binding protein 1 OS = Rattus norvegicus GN = Stxbp1 PE = 1 SV = 1 - [STXB1_RAT]			9.83	3	7.6	3
P18484	AP-2 complex subunit alpha-2 OS = Rattus norvegicus GN = Ap2a2 PE = 1 SV = 3 - [AP2A2_RAT]	0		9.33	3	14.58	4
P62250	40S ribosomal protein S16 OS = Rattus norvegicus GN = Rps16 PE = 1 SV = 2 - [RS16_RAT]	0		9.24	4	12.95	4
P18422	Proteasome subunit alpha type-3 OS = Rattus norvegicus GN = Psma3 PE = 1 SV = 3 - [PSA3_RAT]			9.21	1	6.99	1
P62832	60S ribosomal protein L23 OS = Rattus norvegicus GN = Rpl23 PE = 2 SV = 1 - [RL23_RAT]	0		9.11	2	15.01	3
P04897	Guanine nucleotide-binding protein G(i) subunit alpha-2 OS = Rattus norvegicus GN = Gnai2 PE = 1 SV = 3 - [GNAI2_RAT]	0		8.94	3	8.13	3
“P40329	”“Arginine–tRNA ligase cytoplasmic OS = Rattus norvegicus GN = Rars PE = 1 SV = 2 - [SYRC_RAT]”“				8.7	3	5.28
“P62703	”“40S ribosomal protein S4 X isoform OS = Rattus norvegicus GN = Rps4x PE = 2 SV = 2 - [RS4X_RAT]”“				7.97	3	15.66
P62716	Serine/threonine-protein phosphatase 2A catalytic subunit beta isoform OS = Rattus norvegicus GN = Ppp2cb PE = 1 SV = 1 - [PP2AB_RAT]	0		7.97	2	6.97	3

The table shows the 20 highest scoring hits (out of 334) in two independent samples from hippocampal primary neurons (E17-18) found by pull-down assay with a cross-linking reagent (DTSSP) and magnetic beads covalently linked to HB-GAM (HBGAM_1 and HBGAM_2) and BSA.
