# Supplementary material for: HB-GAM (pleiotrophin) reverses inhibition of neural regeneration by the CNS extracellular matrix
Source: Sci Rep. 2016 Sep 27;6:33916. doi: 10.1038/srep33916 (PMC5037378; doi:10.1038/srep33916)
Supplement: Supplementary Information [file srep33916-s1.pdf]

Supplementary information for the manuscript

HB-GAM (pleiotrophin) reverses inhibition of neural regeneration by the CNS extracellular matrix

Authors: Mikhail Paveliev[1], Keith K. Fenrich[2,3], Mikhail Kislin[1], Juha Kuja-Panula[1], Evgeny Kulesskiy[1], Markku Varjosalo[4], Tommi Kajander[4], Ekaterina Mugantseva[1], Anni Ahonen-Bishopp[1], Leonard Khiroug[1], Natalia Kulesskaya[1], Geneviève Rougon[2] & Heikki Rauvala[1*]

Affiliation: [1] Neuroscience Center, University of Helsinki, Finland, [2] Neuroscience Institute Marseille, France, [3] Neuroscience and Mental Health Institute, Faculty of Rehabilitation Medicine, University of Alberta, Edmonton, Canada, [4] Institute of Biotechnology, University of Helsinki, Finland

*Correspondence: Heikki Rauvala, Address: Neuroscience Center, P.O.Box 56, FIN-00014, University of Helsinki, Finland; telephone: +358 50 3605790; fax: +358 9 191 57620; e-mail: heikki.rauvala@helsinki.fi

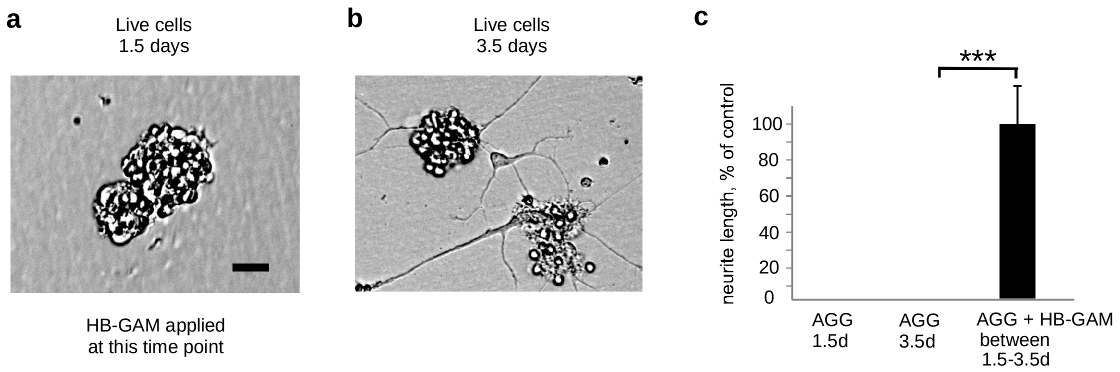

Paveliev et al., Figure S1

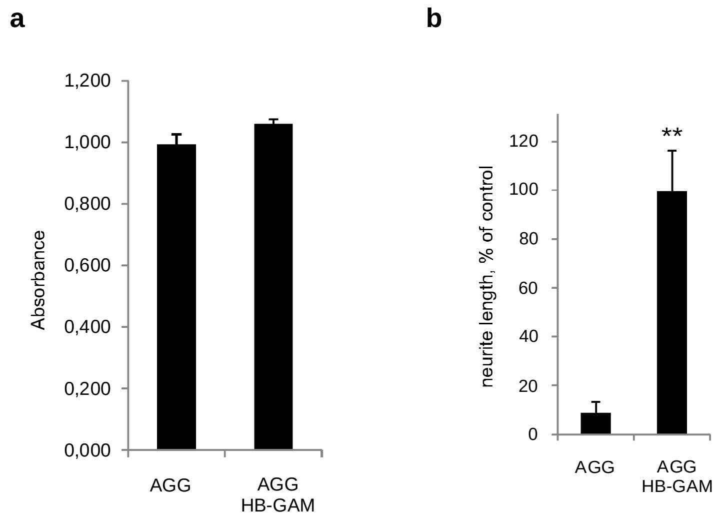

Paveliev et al., Figure S2

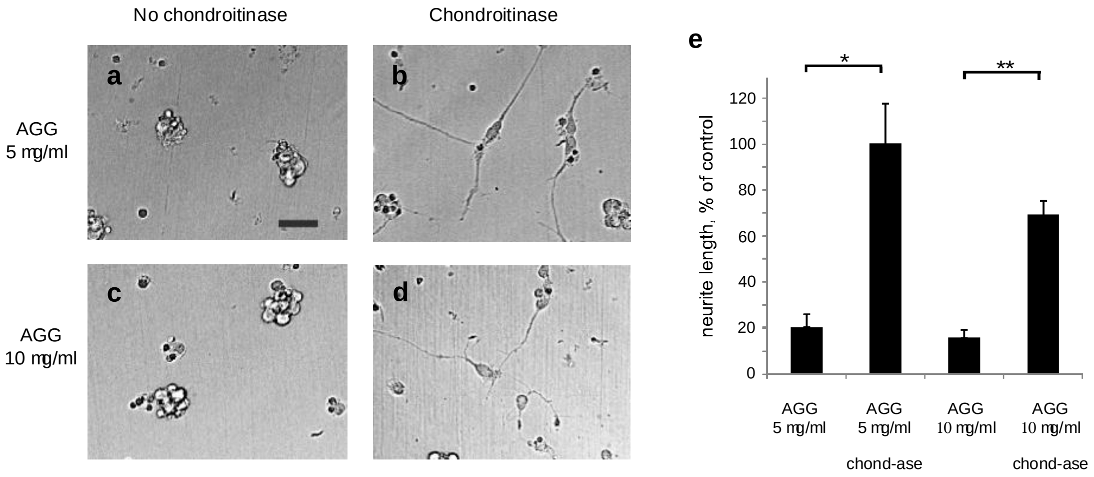

Paveliev et al., Figure S3

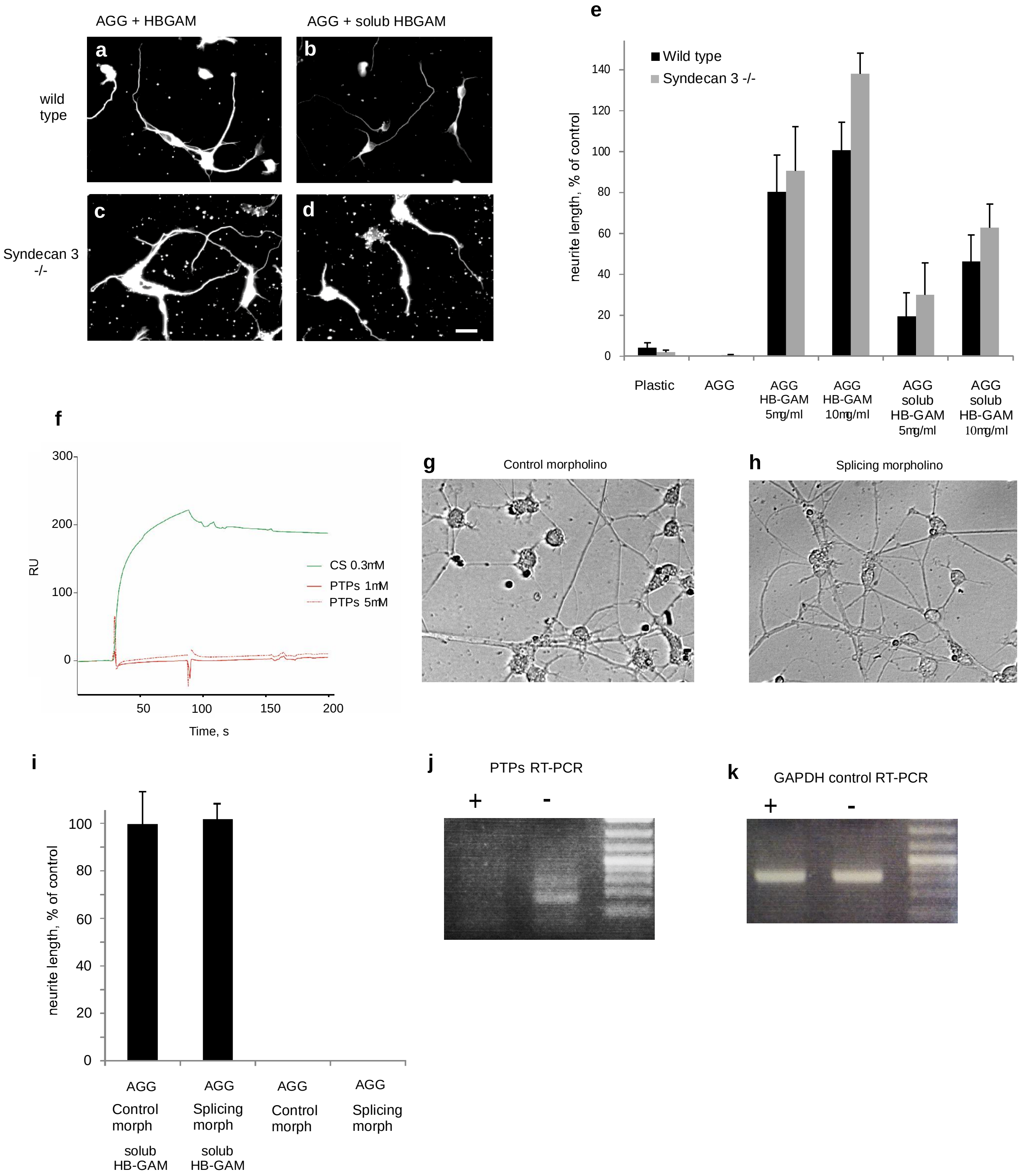

Paveliev et al., Figure S4

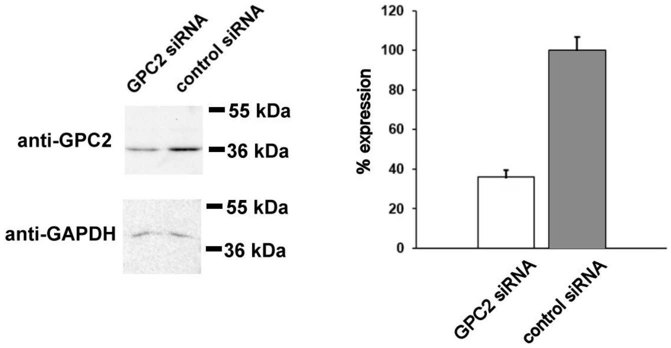

Paveliev et al., Figure S5

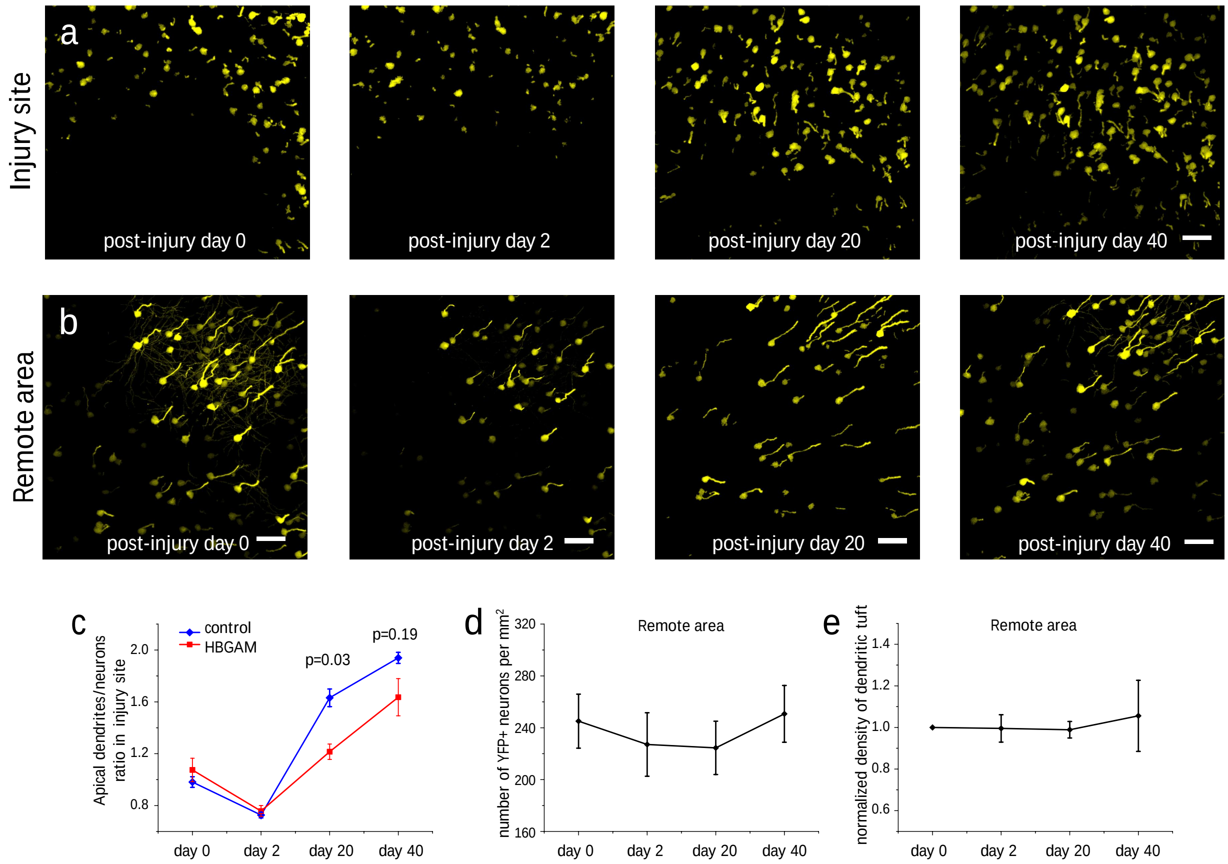

Paveliev et al., Figure S6

Supplementary figure legends

Supplementary Figure 1. Delayed application of HB-GAM overcomes neurite outgrowth inhibition in cortical neurons cultured on a CSPG-rich matrix. (**a-c**) Cortical neurons from E17 rats cultured on substrate precoated with aggrecan (10 µg/ml). HB-GAM (10 µg/ml) was added to culture medium 1.5 days after plating, and the cells were cultured for 2 additional days. One way ANOVA was used in **c**. The scale bar in **a** is 20 µm.

Supplementary Figure 2. HB-GAM does not reduce the amount of coated aggrecan on the substrate. HB-GAM promotes neurite outgrowth in cortical neurons on aggrecan immobilized through biotin-avidin interaction. (**a**) Binding of biotinylated aggrecan to cell culture plastic was measured by colorimetric assay using streptavidin-conjugated HRP after coating in the presence versus absence of HB-GAM (10 µg/ml). (**b**) Biotinylated aggrecan (5 µg/ml) was immobilized on neutravidin plates. Soluble HB-GAM (20 µg/ml) was added when plating neurons.

Supplementary Figure 3. Chondroitinase ABC digestion of the substrate-bound aggrecan decreases its inhibitory effect on neurite outgrowth. (**a**, **c** and **e**) Cortical neurons cultured on the substrate coated with aggrecan at 5 µg/ml or at 10 µg/ml exhibited little or no neurite growth. (**b**, **d** and **e**) In parallel cultures, the aggrecan-coated substrate was treated with chondroitinase ABC (2 U/ml, 30 min) followed by singe wash with PBS. In the chondroitinase ABC-treated samples neurons extended significantly longer neurites compared to the untreated samples. All washing steps were applied equally to the chondroitinase-treated and –untreated samples. The scale bar in **a** (25 µm) is valid for **a**-**d**.

Supplementary Figure 4. The HB-GAM receptor syndecan-3 or the CSPG receptor PTPσ are not required for neurite growth on CSPG in the presence of HB-GAM. (**a-e**) Neurite outgrowth in cortical neurons prepared from P1-P2 syndecan-3 +/+ and -/- mouse littermates. Neurons were cultured on the substrate precoated with aggrecan or with aggrecan + HB-GAM (10 µg/ml each) for 2 days, then fixed and immunostained for tubulin b III. Neurons from syndecan-3 +/+ and -/- mice extend neurites on aggrecan + HB-GAM as shown in **a**, **b** and **e** but not on tissue culture plastic or on the culture plastic coated only with aggrecan as shown in **e**. Soluble HB-GAM induces outgrowth in neurons from both syndecan-3 +/+ and -/- mice on the aggrecan-coated substrate as shown in **c**, **d** and **e**. The scale bar in **d** is 20 µm. (**f**) Surface plasmon resonance was used to test whether the ectodomain of PTPσ (the N-terminal extracellular part that contains three Ig domains, as in Figure 3) interacts directly with HB-GAM. HB-GAM was immobilized on the surface and 1 µM and 5 µM PTPσ (red lines) was injected over it and compared to 0.3 µM shark cartilage CS (green line). All ligands were injected for 60 s. (**g-i**) Neurite outgrowth in E17 cortical neurons cultured for 3 days with the control vivo-morpholino in **g** and **i**, or with the vivo-morpholino inhibiting splicing of PTPσ in **h** and **i** (4 µg/ml each). The cells were cultured on aggrecan (2 µg/ml) for 3 days, then replated on aggrecan (2 µg/ml) with or without HB-GAM (10 µg/ml) in the culture medium and cultured for additional 3 days. (**j** and **k**) Knockdown of the PTPσ mRNA in the neurons is demonstrated by RT-PCR analysis. Neuronal cultures were treated (+) or not treated (-) with the vivo-morpholino inhibiting splicing of PTPσ.

Supplementary Figure 5. Efficiency of glypican-2 siRNAs in reducing the proteoglycan expression. Hippocampal neurons treated with glypican-2 siRNAs and with negative control siRNA were treated with heparinase III to cleave the HS chains in order to facilitate western blotting analysis of the proteoglycan.

Western blotting with anti-glypican-2 antibodies revealed 65% reduction in expression when the cells were treated with the glypican-2 siRNAs. For loading control purposes the same samples were blotted with antibodies against the housekeeping protein GAPDH.

Supplementary Figure 6. HB-GAM improves dendrite regeneration in injury site after prick-injury *in vivo*. The maximum projection (top view) of YFP-labelled layer 5 pyramidal neurons somas (yellow) in injury site (**a**) and remote area (**b**) 3 hours and 2, 20,40 days after injury. **c**. Average ratio of the numbers of apical dendrites per neuron in injury site over time in control experiments (blue line) and following HB-GAM treatment (red line) (p-value from Mann-Whitney-U test). Error bars, SEM (n = 4 control; n = 4 HB-GAM). Number of YFP+ neurons per mm^2 over time in remote area (**d**) and average density of dendritic tufts normalized to the time point of 3h following the injury (**e**) across control and HB-GAM treated animal remain unchanged over time.

Supplementary video legends

Video 1. HB-GAM improves dendrite regeneration in injury site after prick-injury in vivo. In vivo two-photon microscopy revealed robust regeneration of the dendritic tuft and of apical dendrites compared to the start of the experiment (3 h from the acute injury) within 2-3 weeks in the perilesional area in HB-GAM-injected cortical injury sites compared to the controls (Fig. 6c-j; optical sections included in the merged image stacks shown in Video 1).

Video 2. Through-depth videos of spinal cord injury sites immediately following injury from an IgG treated animal (left video) and an HB-GAM treated animal (right video). Each video shows a merged through-depth image stack (c.f., Figures 7E (IgG) and 7H (HB-GAM)) and the individual optical frames used to generate each through-depth image stack. Each frame represents an optical section, with Frame 1 showing the most ventral (i.e., deepest) optical section and the last Frame showing the most dorsal (i.e., most superficial) optical section. Caudal is up, Rostral is down.

Video 3. **Through-depth videos of spinal cord injury sites 14 days after injury from an IgG treated animal (left video) and an HB-GAM treated animal (right video).** Each video shows a merged through-depth image stack (c.f., Figures 7E (IgG) and 7H (HB-GAM)) and the individual optical frames used to generate each through-depth image stack. Each frame represents an optical section, with Frame 1 showing the most ventral (i.e., deepest) optical section and the last Frame showing the most dorsal (i.e., most superficial) optical section. Caudal is up, Rostral is down.

Video 4. Through-depth videos of spinal cord injury sites at 28 days after injury from an IgG treated animal (left video) and an HB-GAM treated animal (right video). Each video shows a merged through-depth image stack (c.f., Figures 7F (IgG) and 7I (HB-GAM)) and the individual optical frames used to generate each through-depth image stack. Each frame represents an optical section, with Frame 1 showing the most ventral (i.e., deepest) optical section and the last Frame showing the most dorsal (i.e., most superficial) optical section. Caudal is up, Rostral is down.
